# The Emerging Role of Exosomes in Diagnosis, Prognosis, and Therapy in Head and Neck Cancer

**DOI:** 10.3390/ijms21114072

**Published:** 2020-06-06

**Authors:** Linda Hofmann, Sonja Ludwig, Julius M. Vahl, Cornelia Brunner, Thomas K. Hoffmann, Marie-Nicole Theodoraki

**Affiliations:** 1 Department of Otorhinolaryngology, Head and Neck Surgery, Ulm University Medical Center, 89075 Ulm, Germany; linda.hofmann@uni-ulm.de (L.H.); Julius.Vahl@uniklinik-ulm.de (J.M.V.); cornelia.brunner@uniklinik-ulm.de (C.B.); t.hoffmann@uniklinik-ulm.de (T.K.H.); 2 Department of Otorhinolaryngology, Head and Neck Surgery, University Hospital Mannheim, 68167 Mannheim, Germany; sonja.ludwig@umm.de

**Keywords:** exosomes, HNSCC, liquid biomarker, tumor microenvironment

## Abstract

Exosomes, the smallest group of extracellular vesicles, carry proteins, miRNA, mRNA, DNA, and lipids, which they efficiently deliver to recipient cells, generating a communication network. Exosomes strongly contribute to the immune suppressive tumor microenvironment of head and neck squamous cell carcinomas (HNSCC). Isolation of exosomes from HNSCC cell culture or patient’s plasma allows for analyzing their molecular cargo and functional role in immune suppression and tumor progression. Immune affinity-based separation of different exosome subsets, such as tumor-derived or T cell-derived exosomes, from patient’s plasma simultaneously informs about tumor status and immune dysfunction. In this review, we discuss the recent understanding of how exosomes behave in the HNSCC tumor microenvironment and why they are promising liquid biomarkers for diagnosis, prognosis, and therapy in HNSCC.

## 1. Introduction

Head and neck squamous cell carcinomas (HNSCC) account for the sixth most common cancer worldwide and are characterized by profound immune suppression. Dysregulated cytokine profiles, impaired activity of effector immune cells, and elevated levels of regulatory immune cells contribute to a highly immune suppressive tumor microenvironment (TME) [1,2,3]. Advanced and recurrent HNSCC have limited therapeutic options, and disease outcome remains poor. Immune therapies aiming to restore patient’s antitumor immune response emerged as promising treatment options for HNSCC [1,3,4]. Antibodies blocking immune checkpoint molecules PD-1 (e.g., pembrolizumab and nivolumab) or CTLA-4 (e.g., ipilimumab) aim to reactivate cytotoxic T lymphocytes [5,6] and are focus of current clinical trials. Yet, only a fraction of patients with recurrent/metastatic and platin-refractory disease [7,8] or platin naïve disease [9] who were treated with PD-1 antibodies showed prolonged remission and improved survival [10].

**Figure 1:**
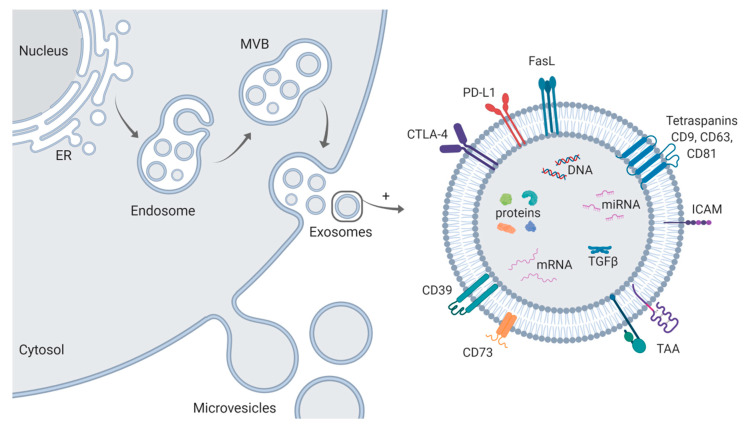
Schematic representation of exosome biogenesis and molecular cargo. Exosomes are formed through inward budding of the endosomal membrane resulting in the formation of multivesicular bodies (MVB). Upon fusion of MVBs with the plasma membrane, exosomes are released in the extracellular space. In contrast, microvesicles are formed by simple budding of the plasma membrane. The molecular cargo of exosomes consists of proteins, miRNA, mRNA, DNA, and lipids. On their surface, they carry the tetraspanins CD9, CD63, and CD81, commonly referred to as “exosomal markers,” adhesion molecules (e.g., intercellular adhesion molecule ICAM) and—in case of TEX—tumor-associated antigens (TAA), which are specific to the cell of origin. Further, the presence of immune suppressive proteins such as CTLA-4, PD-L1, Fas-L, CD39, CD73, and TGFβ in HNSCC-derived exosomes has been reported. Figure is created with BioRender.

Among the mediators contributing to immune suppression in HNSCC, exosomes have become of special interest. Exosomes, the smallest (30–150 nm) of the extracellular vesicles (EVs), are released by all cell types and mediate intercellular communication [11]. Exosomes differ from other EVs by their origin-unique cargo, as their biogenesis process in the endosomal compartment allows them to recapitulate the molecular characteristics of the parental cell [12]. Microvesicles are formed by simple budding of the plasma membrane, whereas exosomes are released upon fusion of the plasma membrane with multivesicular bodies (MVB), which are formed after inward budding of the endosomal membrane (Figure 1) [13]. The molecular cargo of exosomes consists of proteins, miRNA, mRNA, DNA, and lipids [14] and is effectively delivered to recipient cells, generating a communication network. Tumors, including HNSCC, are avid exosome producers and plasma of HNSCC patients is enriched in exosomes [15]. As part of the communication network between tumor cells and immune cells within the TME, exosomes play a major role in immune suppression and the regulation of tumor progression [16,17,18]. Due to their unique biogenesis, their ability to circulate freely in body fluids and their manifold molecular cargo, exosomes have emerged as promising noninvasive liquid biomarkers [19,20]. Several studies showed recently that exosomes have great potential as liquid biomarkers in HNSCC not only for disease activity and tumor stage but also for level of immune suppression and therapy response and outcome [21,22,23,24].

## 2. Isolation and Characterization of Exosomes

**Figure 2:**
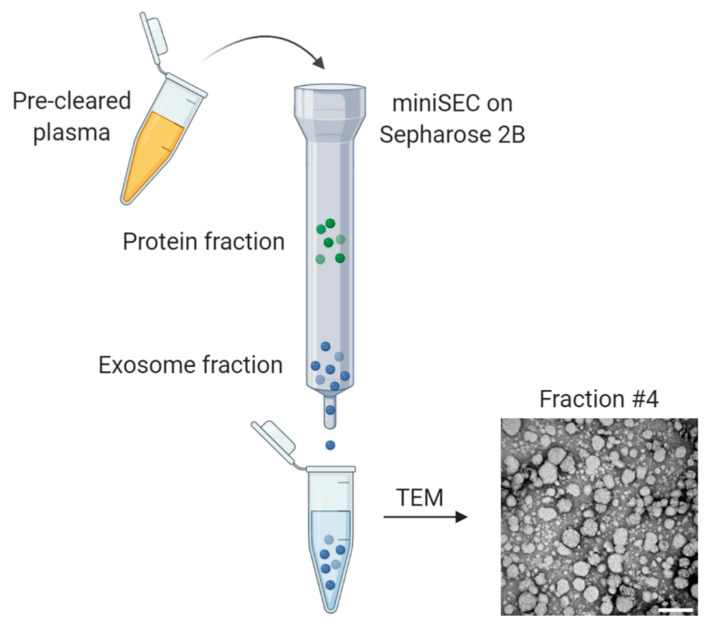
Schematic representation of exosome isolation from plasma using mini-size exclusion chromatography (mini-SEC). Precleared plasma is applied onto a Sepharose 2B column and eluted by serially applying 1 mL PBS. Fraction #4 is enriched in morphologically intact, nonaggregated exosomes as shown in the representative transmission electron microscopy (TEM) picture. Scalebar = 200 nm. Figure is created with BioRender.

Biomarker studies and clinical monitoring with high sample numbers require a fast, high-throughput applicable method for isolation of pure and abundant exosomes. Commonly used methods such as ultracentrifugation, density-gradient centrifugation, or precipitation [25,26] do not fully meet these requirements. Mini-size exclusion chromatography (mini-SEC) has been established and standardized for this purpose (Figure 2) [27]. It enables reproducible isolation of unaggregated exosomes from plasma, which were morphologically and biologically intact as examined in functional coincubation assays with immune cells [27]. To estimate the quality and purity of exosome preparations and to ensure reliability and comparability of studies performed by different investigators, the 2018 minimal information for studies of extracellular vesicles (MISEV) guidelines provide criteria for the definition of EVs and recommendations regarding experimental setups and data interpretation [28]. Accordingly, exosomes need to be characterized for morphology and shape by transmission electron microscopy, size by nanoparticle tracking, and the presence of endosomal markers (such as TSG101) and tetraspanins (CD9, CD63, and CD81) by Western blot [28].

To examine the role of exosomes in HNSCC immune suppression and their potential as liquid biomarkers, the molecular content of exosomes isolated from both cell culture supernatants and plasma has been analyzed by different techniques, and results are presented in the following sections.

## 3. Exosomes Mediate Immune Suppression and Tumor Progression in HNSCC

Although plasma-derived exosomes are a mixture of exosomes derived from different cell types, exosomes from supernatants of tumor cell lines are exclusively tumor-derived (TEX) with no other exosomes present. Thus, the TEX molecular cargo represents a small copy of the parental tumor cell [29]. Much of the current knowledge about the influence of exosomes on the TME was gained by analyzing TEX derived from supernatants of human tumor cell lines (Table 1). Culture conditions of several HNSCC cell lines have been optimized for use with mini-SEC to yield best TEX purity and recovery [30]. Various studies showed that TEX carry immune suppressive proteins and alter the function of recipient immune cells resulting in immune dysfunction [31,32,33]. Even more, TEX were shown to reduce proliferation of CD8+ T cells and induce their apoptosis [31,33]. Simultaneously, TEX promoted expansion, suppressive activity, and resistance of apoptosis of regulatory T cells (Treg) [31,34,35]. TEX-mediated changes on T lymphocytes are described both on transcriptional and functional levels. After incubation with TEX, T cells showed remarkable changes in mRNA expression of various immune response-related genes. These changes translated into reduced CD69 expression on activated T cells and increased production of immune-suppressive adenosine by Treg [32].

Further, TEX were shown to induce tumor innervation [36] and angiogenesis through reprogramming of endothelial cells within the TME [37]. Migration and invasion were induced in a poorly metastatic oral cancer cell line when coincubated with TEX derived from a highly metastatic cell line [38]. Oral squamous cell carcinoma (OSCC)-derived exosomes carrying EGFR transformed normal epithelial cells into a mesenchymal phenotype, and the anti-EGFR therapeutic antibody cetuximab inhibited this carcinogenic effect of TEX [39]. In response to TEX, oral keratinocytes revealed a modulated transcriptome profile that contributed to cancer-associated pathologies such as angiogenesis, immune evasion, and metastasis [40]. Another involvement of TEX has been found in the context of hypoxia, a key factor driving cancer progression and metastasis [41]. As such, TEX derived from hypoxic OSCC cells promoted migration and invasion of normoxic OSCC cells by delivery of miR-21 [42].

Overall, these observations emphasize that TEX-mediated modulation of the TME contributes to immune suppression, tumor growth, and metastasis in HNSCC.

So far, in vivo studies on the effect of systemically delivered TEX on HNSCC tumor progression or immune suppression are rare (Table 2). Early studies with xenograft tumor models showed that OSCC cell-derived exosomes promoted tumor growth in vivo [43]. Further, TEX derived from hypoxic tumor cells induced tumor growth and metastasis in a xenograft model of OSCC [42]. More recently, a 4-nitroquinoline 1-oxide (4NQO) carcinogen-induced orthotopic model of OSCC in C57BL/6 mice was employed [37,44]. The tumorigenic compound 4NQO causes intracellular oxidative stress followed by mutations and DNA strand breaks [45]. The oral mucosa finally undergoes a malignant transformation process that mimics human oral cavity neoplastic transformation in vivo [46,47]. Intravenous TEX administration to 4NQO-conditioned mice at a premalignant stage facilitated disease progression from a premalignant epithelial to a malignant mesenchymal phenotype and reduced the number of tumor-infiltrating lymphocytes [44]. Further, TEX administration resulted in increased vascularization within the tumor and thus promoted angiogenesis in 4NQO-conditioned mice [37].

**Table 1:** Effects of exosomes on the tumor microenvironment: in vitro studies.

Exosome Source	Isolation Method	Outcome	Reference
PCI-13 HNSCC cell line	Differential centrifugation and mini-SEC	TEX induced apoptosis of activated CD8+ T cells and modulated Treg suppressor functions via cell surface signaling.	[33]
PCI-13 HNSCC cell line	SEC and ultracentrifugation	TEX inhibited signaling and proliferation of activated CD8+ T cells and induced expansion of Treg.	[31]
PCI-13 HNSCC cell line	SEC and ultracentrifugation	TEX induced generation, expansion, biologic activity, and resistance to apoptosis of Treg.	[35]
C15 and C17 PDX (patient-derived xenograft) NPC cell line	Differential centrifugation and sucrose gradient flotation	TEX facilitated Treg recruitment and expansion of CD25^high^ FOXP3^high^ Treg.	[34]
PCI-13 HNSCC cell line	Differential centrifugation, SEC, and ultracentrifugation	TEX regulated expression of immune-function related genes in T cell subsets translating into increased adenosine production and loss of CD69 expression on activated T cells.	[32]
UM-SCC-1, UM-SCC-19, UM-SCC-47, and 96-VU-147T-UP-6 HNSCC cell lines	Differential ultracentrifugation and iodixanol gradient centrifugation	TEX and exosomes from patients (both plasma and tumor) stimulated neurite outgrowth in PC12 neuronal model cells.	[36]
PCI-13 and UM-SCC47 HNSCC cell lines	Differential centrifugation and mini-SEC	TEX stimulated proliferation, migration, and tube formation of endothelial cells, thus promoting angiogenesis.	[37]
HOC313 OSCC cell line	SEC and ultracentrifugation	TEX from highly metastatic cells induced cell growth and promoted cell motility of poorly metastatic cells through the delivery of miR-1246.	[38]
HSC-3 and RT-7 OSCC cell lines	Differential centrifugation and Total Exosome Isolation Kit (Invitrogen)	EGFR-positive TEX transformed normal epithelial cells into a mesenchymal phenotype which was inhibited by cetuximab.	[39]
Ca1, CaLH2, SQCC/Y1, SVpgC2a, and SVFN8 OSCC cell lines	Differential centrifugation and ultracentrifugation	TEX changed transcriptome profile in oral keratinocytes regarding pathways involved in matrix remodeling and immune modulation.	[40]
SCC-9 and CAL-27 OSCC cell lines	ExoQuick Exosome Precipitation Kit (System Biosciences)	TEX derived from hypoxic cells increased migration and invasion of normoxic cells by delivery of miR-21.	[42]
HPV(+) UM-SCC-2, UM-SCC-47, UPCI-SCC-90, HPV(−) PCI-13, and PCI-30 HNSCC cell lines	Differential centrifugation and mini-SEC	HPV(+) and HPV(−) TEX carried immune modulatory proteins and inhibited T cell function. Only HPV(−) TEX suppressed dendritic cell function.	[48]
HPV(+) UM-SCC-2, UM-SCC-47, UPCI-SCC-90, HPV(−) PCI-13, and PCI-30 HNSCC cell lines	Differential centrifugation and mini-SEC	The proteomic cargo differed between HPV(+) and HPV(−) TEX. HPV(+) TEX were enriched in CD47 and CD276, whereas HPV(−) TEX contained tumor-protective/growth-promoting antigens, MUC-1 and HLA-DA.	[49]
HPV(+) SCC-90, SCC-47, SCC-104, HPV(−) SAS, CAL-27, and CAL-33 HNSCC cell lines	Differential centrifugation and ultracentrifugation	MiR-9-enriched TEX from HPV(+) HNSCC transformed macrophages into the M1 phenotype and increased the radiosensitivity of HPV(+) HNSCC.	[50]
HSC-3 and SCC-9 OSCC cell lines	Differential centrifugation and ultracentrifugation	TEX derived from cisplatin-resistant cells induced chemoresistance in platin-naive cells and decreased DNA damage signaling in response to cisplatin.	[51]
Primary, HNSCC patient-derived cancer-associated fibroblasts	Differential centrifugation and ultracentrifugation	TEX derived from cisplatin-resistant cancer-associated fibroblasts conferred chemoresistance and an aggressive phenotype in cancer cells by transfer of functional miR-196a.	[52]
KYSE30, KYSE70, and KYSE180 ESCC cell lines	Differential centrifugation and ultracentrifugation	Radioresistant cells showed a differential miRNA expression profile compared to normal cells and exosomal miR-339-5p mediated regulation of radiosensitivity.	[53]
UM-SCC-6 HNSCC cell line	Differential centrifugation and SEC	Proteomic analysis of TEX released from irradiated cells revealed overexpressed proteins involved in response to radiation, ROS metabolism, and DNA repair.	[54]
FaDu HNSCC cell line	Total Exosome Isolation Kit (Invitrogen) and ultracentrifugation	Proteomic profile of TEX released from irradiated cells was significantly altered compared to TEX from nonirradiated cells.	[55]
BHY and FaDu HNSCC cell lines	Differential centrifugation and ultracentrifugation	TEX derived from irradiated cells promoted survival and proliferation and conferred a migratory phenotype to recipient cancer cells.	[56,57]

These findings confirm the pathophysiological role of TEX in HNSCC tumorigenesis and disease progression in vivo and are summarized in Table 2. In addition, the 4NQO model represents a suitable tool for further investigation of TEX-driven carcinogenesis in vivo.

## 4. Molecular and Functional Profiles of Exosomes from HPV(+) and HPV(−) Tumors

Infection with human papillomavirus (HPV) belongs to the main etiologic risk factors for HNSCC, especially in the oropharynx with an HPV prevalence of around 25% [58,59,60]. Clinical, histopathological and molecular characteristics are different between HPV(+) and HPV(−) HNSCC [61]. HPV(+) tumors are generally more responsive to therapy and have a better prognosis and outcome with an approximately 60% reduced risk of death compared to HPV(−) tumors [62,63,64]. TEX from HPV(+) and HPV(−) HNSCC cell lines were analyzed regarding their differential capabilities to modulate the antitumor immune response [48,49]. Both HPV(+) and HPV(−) TEX carried immunomodulatory molecules. However, only HPV(+) TEX promoted immune activity of dendritic cells by driving their maturation and the expression of antigen-processing machinery components on their surface [48]. Additionally, comparison of proteome profiles by mass spectrometry revealed differential content of protein cargos in HPV(+) and HPV(−) TEX [49]. The presence of CD47, a supposed antiphagocytic molecule, on HPV(+) TEX might support prolonged interactions with immune cells [49]. Recently, HPV(+) TEX were found to foster M1 polarization of macrophages via miR-9, which may contribute to radiosensitivity of HNSCC [50].

Overall, HPV(+) and HPV(−) TEX are supposed to differentially modulate antitumor immune response thereby playing a role in disease progression and outcome. Hence, HPV(+) TEX might promote antitumor immune response thereby improving outcome of patients with HPV(+) cancers.

## 5. Exosomes as Biomarkers for Disease Progression and Activity

Studies with exosomes isolated from plasma of HNSCC patients are a prerequisite to establish their role as liquid biomarkers. HNSCC patients were shown to have significantly higher exosome levels compared to healthy donors [15,27]. Further, the exosomal protein concentration and molecular content are correlated with disease activity and tumor stage. Patients with active disease (AD) or Union for International Cancer Control (UICC) high stage had significantly higher exosome levels and higher levels of immune-suppressive molecules carried by these exosomes compared to patients with nonevident disease (NED) or UICC low stage [15,21]. Consistent with the immune modulatory characteristics of cell line-derived TEX, plasma-derived exosomes were shown to interfere with immune cells as they suppressed activation and proliferation of T lymphocytes [27]. Even more, exosomes derived from patients with AD were significantly more effective in inducing apoptosis of CD8+ T cells, suppression of CD4+ T cell proliferation, and induction of Treg activity than exosomes from patients with NED, thereby mediating stronger immune suppression [15]. Overall, plasma-derived total exosomes can distinguish between healthy donors and HNSCC patients as well as between low- and high-stage HNSCC.

The PD-1/PD-L1 pathway is an important immune suppressive mechanism operating in the TME and presenting a promising drug target for antibody-based immune therapies in HNSCC [1,3,4]. Yet, resistance to PD-1 blockade therapy is frequent. Exosomes derived from plasma of HNSCC patients were found to carry biologically active PD-L1, which suppressed function of activated T cells [21]. Relative levels of PD-L1 on exosomes were associated with disease activity, clinical stage, and the presence of lymph node metastasis. Patients whose exosomes showed high levels of PD-L1 had active or advanced disease. Further, exosomes with high levels of PD-L1 strongly suppressed T cell activity, whereas exosomes with low PD-L1 levels did not [21]. This immune-suppressive effect was almost completely reversed by adding an anti-PD-1 antibody to the T cell cultures prior to incubation with exosomes. In contrast to exosomal PD-L1, soluble PD-L1 levels did not correlate with clinicopathological data [21]. These findings can be explained by increased protein degradation of the unprotected soluble PD-L1 in contrast to stable PD-L1 integrated in the exosomal membrane. Thus, PD-L1 on exosomes emerged as a promising biomarker for disease progression of HNSCC. Similar correlations of exosomal PD-L1 with immune suppression and tumor growth have been later reported for other tumor entities, such as melanoma or lung cancer [65,66,67]. In future, the influence of PD-L1-positive exosomes on immune therapy and resistance needs to be evaluated.

Exosomal miRNAs are supposed to have significant functions in the regulation of cancer progression [68]. miR-21 has been identified as a common proto-oncogene and its target genes are involved in several processes controlling carcinogenesis, such as proliferation, apoptosis, and invasion [69]. Patients with OSCC were found to have significantly higher levels of serum exosomal miR-21 compared to healthy volunteers [42]. Further, exosomal miR-21 levels correlated with T stage and lymph node metastasis as well as with the tumor HIF-1α/2α expression, reflecting the hypoxic status of the tumor [42]. Similar studies revealed that miR-21 in serum-derived exosomes correlated with advanced tumor stage and metastasis in laryngeal (LSCC) [70] and esophageal squamous cell carcinoma (ESCC) [71]. These studies emphasized the clinical impact of exosomal miR-21 as a valuable biomarker for HNSCC progression.

Recently, a quantitative proteomics approach was applied to identify the protein content of serum-derived exosomes in OSCC patients with or without evidence of lymph node metastasis and compared to healthy controls [72]. Thereby, PF4V1, CXCL7, F13A1, and ApoA1, proteins involved in regulation of metastasis and cancer progression, were found to be differentially expressed and correlated to tumor differentiation level and metastasis. Receiver operating characteristic (ROC) curve analysis indicated that a combination of different biomarkers improved diagnostic accuracy compared to a single biomarker [72].

## 6. TEX and Non-TEX as Biomarkers for Tumor Status and Immune Dysfunction

Capture techniques based on immune affinity allow for the separation of exosomes according to their origin or presence of tumor-specific epitopes on their surface. This way, distinct exosome subsets can be analyzed regarding their potential as biomarkers. Using antibodies against chondroitin sulfate proteoglycan 4 (CSPG4), a tumor antigen selectively expressed by melanoma and other malignant but not normal cells [73,74], melanoma-derived TEX were successfully isolated from patients’ plasma [75]. However, the molecular heterogeneity of HNSCC makes it difficult to identify markers for this tumor entity. A mix of antibodies recognizing antigens commonly overexpressed on HNSCC (EGFR, MAGEA3, EpCAM, and CSPG4) has been used for the construction of a microarray for TEX capture from plasma [22]. More recently, CD44v3 was evaluated as a tumor-associated protein to selectively enrich TEX from plasma of HNSCC [76]. CD44v3 overexpression has been linked to tumor progression and metastatic potential in HNSCC [77,78,79]. The molecular profile of CD44v3(+) TEX was strongly immune suppressive and correlated with higher disease stage and lymph node metastasis. Thus, CD44v3(+) TEX represented a potential biomarker of HNSCC activity and progression. A similar immune-capture approach targeting CD45 on plasma-derived exosomes enabled separation of CD45(+) hematopoietic cell-derived exosomes and CD45(−) TEX-enriched exosomes [76].

Exosomes present in plasma of HNSCC patients resemble a mix of TEX and non-TEX. Although TEX might serve as biomarkers for tumor status, exosomes produced from immune cells can serve as biomarkers for immune dysfunction [20,80]. Immune affinity-based capture with CD3 antibodies was used to separate T cell-derived CD3(+) exosomes from CD3(−) exosomes [23,81]. CD3(−) exosomes were CD44v3 positive and thus largely tumor derived. Both exosome subsets carried immune regulatory proteins such as PD-L1, CTLA-4, COX-2, or CD15s and induced apoptosis of activated T cells [23]. The cargo of both CD3(+) and CD3(−) exosomes correlated with tumor stage and nodal status albeit the associations were weaker for the CD3(−), tumor-enriched fraction. Patients with high-stage disease or lymph node metastasis had higher levels of immune-suppressive and lower levels of immune-stimulatory markers compared to patients with low-stage disease or absence of lymph node metastasis. CD3(−) exosomes from patients with advanced disease carried the highest levels of enzymatically active CD39 and CD73 [81]. These exosomes spontaneously produced immune-suppressive adenosine in the presence of exogenous adenosine triphosphate (ATP) and induced adenosine production in Treg, as previously reported for total exosomes from plasma of HNSCC patients [82]. In contrast, CD3(+) exosomes from patients with early disease carried significantly higher levels of adenosine deaminase (ADA) and CD26, involved in adenosine degradation, compared to CD3(+) exosomes from patients with advanced disease, indicating that the latter bear higher immune suppression [81]. By separating T cell-derived CD3(+) and TEX-enriched CD3(−) exosome subsets, simultaneous assessment of immune cell competence and tumor status as well as tumor-induced immune suppression was feasible.

In HNSCC, altered natural killer (NK) cell functions strongly contribute to the immune suppressive, protumorigenic TME [83,84,85]. NK cells express the Fc receptor CD16, hence they are able to participate in antibody-dependent cell-mediated cytotoxicity (ADCC) [86]. Recent data showed that CD16 is also present on exosomes derived from plasma of HNSCC patients, and CD16 levels were higher on total exosomes compared to TEX [87]. Further, CD16 levels on total exosomes but not TEX correlated with tumor stage and tumor aggressiveness. Patients with high and advanced tumor stages had significantly higher CD16 levels on total exosomes compared to low-stage patients. CD16-positive exosomes emerged as mediators of immune suppression as they could mimic NK cells in their function of cross-linking with antibody-coated malignant cells without implementing their cytotoxic function. Further, CD16-positive exosomes might reduce the efficacy of antibody therapies by serving as antibody-decoy as described for immune checkpoint inhibition with trastuzumab (anti-HER2 receptor monoclonal antibody) [88].

**Figure 3:**
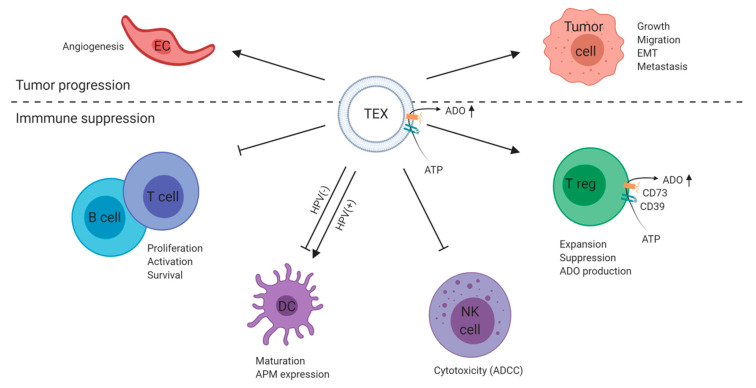
Summary of tumor-derived exosomes (TEX) interactions in head and neck squamous cell carcinomas (HNSCC). TEX produced by parental tumor cells mediate intercellular communication and play a key role in tumor progression and immune suppression. TEX induce angiogenesis by reprogramming of endothelial cells (EC) [37] and growth, migration, and metastasis of parental tumor cells [38,39,40,42]. Further, TEX alter the function of recipient immune cells resulting in immune dysfunction. They reduce lymphocyte proliferation and induce lymphocyte apoptosis [15,23,27,31,32,33], alter maturation of dendritic cells (DCs) and the expression of antigen-processing machinery components on DCs depending on the TEX HPV profile [48,49], and induce suppression of cytotoxicity in natural killer (NK) cells [15,27]. TEX carry enzymatically active CD39 and CD73 on their surface, thus being able to produce immune-suppressive adenosine [15,81,82]. Additionally, TEX promote expansion, suppressive activity, and adenosine production in regulatory T cells (Treg) [31,32,33,34,35]. Figure is created with BioRender.

**Figure 4:**
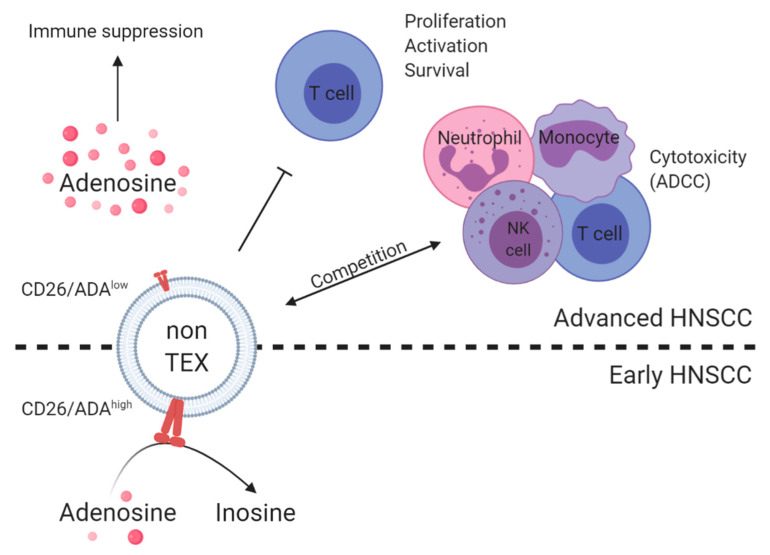
Summary of non-TEX interactions in HNSCC. Non-TEX, mainly immune cell-derived exosomes, contribute to the immune suppression in HNSCC and have a dual role in early versus advanced disease. Although non-TEX from plasma of patients with early HNSCC have high levels of CD26 and adenosine deaminase (ADA), which degrade immune-suppressive adenosine, non-TEX from plasma of patients with advanced HNSCC have low levels of CD26 and ADA resulting in high levels of immune-suppressive adenosine in those patients [81]. Further, the cargo of non-TEX correlated with advanced HNSCC regarding the inhibition of effector T cells [23] and the presence of CD16, which presumably competes with immune cells for antibody-dependent cellular cytotoxicity (ADCC) [87]. Figure is created with BioRender.

A summary of TEX and non-TEX interactions involved in tumor progression and immune suppression is presented in Figure 3; Figure 4.

## 7. Exosomes as Biomarkers and Players in Response to Therapy and Outcome

Evidence from studies on cell-line-derived TEX (Table 1) suggests the involvement of exosomes in the effective treatment of HNSCC, including resistance to chemo- and radiotherapy. In particular, TEX derived from cisplatin-resistant OSCC cell lines were found to induce chemoresistance in platin-naive OSCC cells and decrease DNA damage signaling in response to cisplatin [51]. Similarly, TEX derived from cisplatin-resistant cancer-associated fibroblasts conferred chemoresistance and an aggressive phenotype in HNSCC cells by transfer of functional miR-196a [52]. Radioresistant ESCC cells were found to show differential miRNA expression profile and exosomal miR-339-5p was supposed to mediate regulation of radiosensitivity [53]. Further, the proteomic profile of TEX released from irradiated HNSCC cells was significantly altered compared to TEX from nonirradiated cells [54,55]. Overexpressed proteins were assigned to cell division, DNA repair, and metabolism of radical oxygen species, indicating that the proteomic profile of TEX released by irradiated cells reflects radiation-induced changes of cellular processes [54,55]. Even more, TEX derived from irradiated HNSCC cells promoted proliferation and conferred a migratory phenotype to recipient cancer cells [56,57]. Overall, these studies on cell-line derived TEX indicate that exosomes play a functional role in the response of tumor cells to chemo- and radiotherapy.

Rodrigues-Junior et al. analyzed the ability of exosomes to predict therapy outcome by analyzing pooled plasma samples from locally advanced HNSCC patients who had complete or incomplete response to chemoradiation therapy [89]. They identified a different proteomic profile between exosomes derived from responders and nonresponders. In chemoradiosensitive responders, proteins clustered in pathways related to FAS, p53, and apoptosis signaling. In chemoradioresistant nonresponders, proteins clustered in pathways related to tumorigenesis and angiogenesis pathways. These findings suggest that the content of circulating plasma-derived exosomes has a relevant function in the treatment response of HNSCC patients.

The role of exosomes and their molecular cargo for monitoring patient’s response to therapy has been studied in a small cohort of patients with recurrent, therapy-refractive HNSCC undergoing photodynamic therapy (PDT) [24]. PDT is a palliative treatment option in which, after accumulation of a photosensitizer in the tumor, light activation induces a photochemical reaction with the production of cytotoxic reactive oxygen species. This results in tumor cell damage, local inflammation, and activation of innate and adaptive immune responses with the long-term development of an antitumor immunity [90,91]. Exosomes isolated from plasma of patients treated with PDT at different time points before and after therapy were analyzed regarding their potential to regulate epithelial-mesenchymal transition (EMT), a process conversing tumors from an epithelial to a highly aggressive and invasive mesenchymal phenotype [92]. Before therapy, exosomes from all patients showed a strong mesenchymal profile. However, after therapy, levels of the mesenchymal marker N-cadherin decreased, whereas levels of the epithelial marker E-cadherin showed a significant increase. Furthermore, the known EMT inducer TGF-β was significantly reduced in exosomes after PDT. These exosomes were also able to either promote or suppress EMT in recipient tumor cells dependent on the time point of exosome harvesting before or after PDT. This dual potential of exosomes to modulate EMT in a TGF-β related manner suggested that exosomes contributed to the mesenchymal to epithelial transition of the tumor responding to PDT.

A recent study evaluated the predictive value of TEX and T cell-derived exosomes (separated using CD3 capture) on therapy response in HNSCC patients treated with cetuximab, ipilimumab, and radiation in a Phase I trial [22]. Exosomes were used to discriminate between patients whose disease recurred within 2 years and patients who remained disease-free. The TEX/total exosome ratio was assessed using the abovementioned microarray containing an antitumor antibody cocktail. Only patients with recurrence showed a significant increase of TEX levels after therapy compared to baseline, whereas disease-free patients had reduced TEX levels. Further, levels of CD3(+) exosomes remained unchanged throughout therapy in disease-free patients, whereas they were elevated during therapy in patients with recurrence. CD3(+)15s(+) Treg-derived exosomes seemed to contribute to this increase of CD3(+) exosomes. Overall, these data suggest that exosomes can serve as biomarkers for monitoring patients’ response to oncological therapy.

Until now, therapy-induced changes in exosomes have largely been studied using in vitro cell lines, and only a few studies were performed with exosomes isolated from plasma of patients. Despite the small patient cohorts, these results are promising and larger patient cohorts need to be investigated to validate exosomes as biomarkers for therapy response and outcome.

Table 3 provides an overview of the presented studies on plasma or serum-derived exosomes of HNSCC patients and highlights the analyzed exosome subsets and biomolecules as well as the outcome of the study.

## 8. Exosomes as Therapeutic Vesicles

Patients with metastatic or recurrent HNSCC often do not respond to conventional therapies or develop drug resistance. Targeted delivery of chemotherapeutics might increase the effectiveness of such treatments, prevent chemoresistance, and decrease cytotoxic side effects. Exosomes, as endogenous nanocarriers for various molecules, are emerging as drug delivery vehicles both for chemotherapeutics as well as therapeutic short interfering RNAs (siRNA) due to their low immunogenicity, strong ability to cross physiological barriers, good biodistribution, and bioavailability [93,94,95]. Their potential for drug delivery has been examined in several tumor entities such as pancreas carcinoma [96,97] and breast cancer [98,99], whereas studies on HNSCC are rare. Exosomes loaded with the chemotherapeutics doxorubicin or paclitaxel were shown to accumulate efficiently in target tumor tissues and inhibit tumor growth in a breast cancer mouse model without overt side effects [98,99]. In another study, incorporation of paclitaxel in exosomes increased its cytotoxicity against multidrug-resistant cancer cells [100], indicating the possibility to overcome drug resistance by the use of exosome-encapsulated chemotherapeutics. Exosomes carrying siRNAs specific to oncogenic *Kras* have been shown to suppress cancer in multiple mouse models of pancreatic cancer, significantly increasing overall survival [97]. The same study revealed enhanced retention of these exosomes, compared to liposomes, in the circulation of mice due to reduced clearance by the mononuclear phagocyte system. In fact, exosomes have already undergone clinical trials in melanoma [101], colorectal [102], and lung cancer [103,104]. Recent data showing that exosome-delivered miRNA-138 efficiently conferred its OSCC antitumor functions in vitro and in vivo support the presumption that exosomes have potential as delivery agents also in HNSCC [105]. Further, engineered exosomes have been considered as therapeutic anticancer vaccines for HPV-associated tumors [106]. This approach is based on the mutant HIV-1 negative regulatory factor (Nef^mut^) protein, which remarkably incorporates into exosomes and acts as an exosome-anchoring protein upon fusion with heterologous proteins [107,108]. Upon inoculation in mice, Nef^mut^/HPV-E7 exosomes induced an E7-sepcific cytotoxic T lymphocyte (CTL) immune response [109]. Even more, intramuscular immunization of mice with a DNA vector encoding Nef^mut^ fused to HPV-E7 provided the animals with a source of endogenously engineered EVs, induced an E7-specific CTL activity, and blocked growth of syngeneic tumor cells in immunized mice bearing subcutaneous HPV-positive tumors [110].

Given these intriguing findings, extensive investigations such as evaluation of exosome pharmacokinetics and quantitative analysis in biological fluids [111] are required and ongoing to implement exosome-based drug delivery for HNSCC.

## 9. Conclusion and Outlook

The unique molecular cargo of exosomes, either tumor or immune cell-derived, allows to alter the function of recipient cells. The diverse exosome-mediated changes in the TME contribute to tumor progression and immune suppression. Due to the importance of exosomes in HNSCC carcinogenesis and antitumor immune response, exosomes may serve as potential biomarkers of diagnosis, prognosis, and therapy response in HNSCC. Although first studies on exosomes isolated from patient’s plasma show promising results, there is a need to validate the diagnostic and prognostic profile of exosomes in large patient cohorts and clinical trials. Further, studies need to explore the clinical application as therapeutic vesicles in HNSCC.

## Figures and Tables

**Table 2:** Effects of exosomes on tumor progression and immune suppression: in vivo studies.

Exosome Source	Isolation Method	Mouse Model	Outcome	Reference
OSC-4 OSCC cell line	Total Exosome Isolation Kit (Invitrogen)	OSC-4 xenografts implanted into nude mice	TEX promoted growth rate of tumor xenografts, which could be inhibited by continuous administration of heparin.	[43]
SCC-9 and CAL-27 OSCC cell lines	ExoQuick Exosome Precipitation Kit (System Biosciences)	CAL-27 xenografts implanted into nude mice	Tumor-derived exosomal miR-21 induced tumor growth and metastasis in a xenograft OSCC model.	[42]
PCI-13 and UM-SCC-47 HNSCC cell lines	Differential centrifugation and mini-SEC	4-NQO oral carcinogenesis mouse model	TEX promoted formation of defined vascular structures within the tumor and thus, promoted angiogenesis.	[37]
SCCVII, SCC-90, and PCI-13 HNSCC cell lines	Differential centrifugation and mini-SEC	4-NQO oral carcinogenesis mouse model	TEX promoted tumor progression and reduced immune cell migration to the tumor.	[44]

**Table 3:** Exosome studies involving patients.

Exosome Source	Isolation Method	Exosome Subset	Methods	Molecules	Outcome	Prediction	Reference
Plasma of HNSCC patients	Differential centrifugation and mini-SEC	Total exosomes	Nanoparticle tracking, western blot, functional coincubation assays	- (Establishment of mini-SEC)	Mini-SEC allows for simple and reproducible isolation from human plasma of exosomes retaining structural integrity and functional activity.	–	[27]
Plasma of HNSCC patients, *n* = 38	Differential centrifugation and mini-SEC	Total exosomes	Western blot, functional coincubation assays	- (Exosome-mediated immune suppression)	Patients with active disease (AD) had significantly higher exosome levels compared to patients with nonevident disease (NED). Exosomes from patients with AD mediated stronger immune suppression than exosomes from patients with NED.	Tumor progression/disease activity and immune status	[15]
Plasma of HNSCC patients, *n* = 40	Differential centrifugation and mini-SEC	Total exosomes	On-bead flow cytometry, and functional coincubation assays	PD-L1	Levels of PD-L1 on exosomes correlated with disease activity, UICC stage, and the presence of lymph node metastasis. In contrast, plasma levels of soluble PD-L1 did not correlate with any clinicopathological data. High PD-L1 levels, but not low PD-L1 level, exosomes suppressed T cell activity, which could be attenuated with an anti-PD-1 antibody.	Tumor progression/disease activity	[21]
Plasma of OSCC patients, *n* = 108	ExoQuick Exosome Precipitation Kit (System Biosciences)	Total exosomes	miRNA sequencing	miR-21	Exosomal miR-21 levels correlated with advanced T classification, the presence of lymph node metastasis, and tumor HIF-1α/2α expression.	Tumor progression/disease activity	[42]
Serum of LSCC patients, *n* = 52	ExoQuick Exosome Precipitation Kit (System Biosciences)	Total exosomes	miRNA analysis (RT-PCR)	miR-21	Exosomal miR-21 and HOTAIR levels correlated with advanced T classification and UICC high stage.	Tumor progression/disease activity	[70]
Serum of ESCC patients, *n* = 51	ExoQuick Exosome Precipitation Kit (System Biosciences)	Total exosomes	miRNA analysis (RT-PCR)	miR-21	Exosomal miR-21 levels correlated with advanced T classification, positive lymph node status, and the presence of metastasis.	Tumor progression/disease activity	[71]
Serum of OSCC patients, *n* = 30	ExoQuick Exosome Precipitation Kit (System Biosciences)	Total exosomes	Quantitative proteomics approach and bioinformatics	PF4V1, CXCL7, F13A1, and ApoA1	PF4V1, CXCL7, F13A1, and ApoA1 were correlated to tumor differentiation level, the presence of lymph node metastasis, and the abusus of alcohol and tobacco. Combining these biomarkers improved diagnostic accuracy compared to a single biomarker.	Tumor progression/disease activity	[72]
Plasma of HNSCC patients, *n* = 44	Differential centrifugation and mini-SEC	Total exosomes, T cell exosomes (CD3 separation), and TEX (CD44v3 capture)	On-bead flow cytometry	CD44v3	CD44v3 levels on CD3(−) exosomes were higher in patients than in healthy donors and correlated with UICC stage and lymph node metastasis. The molecular profile of CD44v3(+) exosomes was strongly immune-suppressive and correlated with disease stage and lymph node metastasis.	Tumor progression/disease activity	[76]
Plasma of HNSCC patients, *n* = 22	Differential centrifugation and mini-SEC	T cell exosomes and TEX (CD3 separation)	On-bead flow cytometry and functional coincubation assays	PD-L1, CTLA-4, COX-2, and CD15s	CD3(+) and CD3(−) exosomes carried immune regulatory proteins and induced apoptosis of activated T cells. The cargo of both subsets correlated with tumor stage and nodal status albeit the associations were weaker for the CD3(−) fraction.	Tumor progression/disease activity	[23]
Plasma of HNSCC patients, *n* = 14	Differential centrifugation and mini-SEC	T cell exosomes and TEX (CD3 separation)	On-bead flow cytometry, functional coincubation assays, and mass spectrometry	CD39, CD73, ADA, CD26, and adenosine	High CD39/CD73 levels and adenosine production were found in patients with UICC high stage. ADA/CD26 levels on CD3(+) exosomes correlated with UICC low stage.	Tumor progression/disease activity and immune status	[81]
Plasma of HNSCC patients, *n* = 14	Differential centrifugation, SEC, and ultracentrifugation	Total exosomes	Mass spectrometry and functional coincubation assays	CD39 and CD73	Exosomes carried enzymatically active CD39 and CD73 and, when supplied with exogenous ATP, hydrolyzed it to adenosine.	Immune status	[82]
Plasma of HNSCC patients, *n* = 53	Differential centrifugation and mini-SEC	Total exosomes and TEX (CD44v3 capture)	On-bead flow cytometry	CD16	CD16 on total exosomes but not TEX, correlated with advanced T classification and UICC high stage.	Tumor progression/disease activity	[87]
Plasma of HNSCC patients undergoing chemoradiation therapy (CRT), *n* = 12	Beads coated with cholera toxin chain B (CTB) and annexin V (AV)	CTB- and AV-exosomes	Antibody array	List of potential markers analyzed by the array	Exosomes from responders and nonresponders to CRT showed a different proteomic profile. Differentially present proteins in exosomes from responders and nonresponders were associated to FAS, p53, and apoptosis pathways or tumorigenesis and angiogenesis, respectively.	Therapy response/outcome	[89]
Plasma of HNSCC patients undergoing photodynamic therapy (PDT), *n* = 9	Differential centrifugation and mini-SEC	Total exosomes	On-bead flow cytometry and functional coincubation assays	EMT-associated markers (TGFβ, E-cadherin, and N-cadherin)	Exosomes harvested before PDT had a mesenchymal profile and enhanced tumor proliferation, migration, and invasion. In contrast, exosomes harvested after PDT had an epithelial profile, restored the epithelial morphology of tumor cells, and inhibited their proliferation, migration, and invasion.	Therapy response/outcome	[24]
Plasma of HNSCC patients enrolled in a phase I clinical trial and receiving cetuximab, ipilimumab, and radiation, *n* = 18	Differential centrifugation and mini-SEC	T cell exosomes and TEX (CD3 separation)	On-bead flow cytometry and antibody microarray	PD-L1, CTLA-4, and CD15s	In recurrent patients, TEX levels, total CD3(+), CD3(−) PD-L1+, and CD3(+) CD15s+ (Treg-derived) exosomes increased from baseline levels. In disease-free patients, TEX levels decreased, CD3(+) and CD3(+) CD15s+ exosomes stabilized and CD3(+) CTLA4+ exosomes declined after ipilimumab therapy.	Therapy response/outcome and disease recurrence	[22]

## Abbreviations

AD	Active disease
ADA	Adenosine deaminase
ADCC	Antibody-dependent cell-mediated cytotoxicity
ATP	Adenosine triphosphate
CSPG4	Chondroitin sulfate proteoglycan 4
CTL	Cytotoxic T lymphocyte
DC	Dendritic cell
EC	Endothelial cell
EMT	Epithelial-mesenchymal transition
ESCC	Esophageal squamous cell carcinoma
EV	Extracellular vesicle
HNSCC	Head and neck squamous cell carcinoma
HPV	Human papilloma virus
LSCC	Laryngeal squamous cell carcinoma
MISEV	Minimal information for studies of extracellular vesicles
MVB	Multivesicular body
NED	Nonevident disease
Nef	Negative regulatory factor
NK	Natural killer
OSCC	Oral squamous cell carcinoma
PDT	Photodynamic therapy
ROC	Receiver operating characteristic
SEC	Size exclusion chromatography
siRNA	Short interfering RNA
TEM	Transmission electron microscopy
TEX	Tumor-derived exosomes
TME	Tumor microenvironment
Treg	Regulatory T cells
UICC	Union for International Cancer Control
4NQO	4-nitroquinoline 1-oxide
